# Population meeting surveillance-based thresholds for metabolic and bariatric surgery evaluation under the 1991 NIH and 2022 ASMBS/IFSO criteria across 37 settings: STEPS analysis and projections to 2030

**DOI:** 10.1016/j.obpill.2026.100329

**Published:** 2026-08-29

**Authors:** Víctor Juan Vera-Ponce

**Affiliations:** Facultad de Medicina (FAMED), Universidad Nacional Toribio Rodríguez de Mendoza de Amazonas (UNTRM), Amazonas, Peru

**Keywords:** Bariatric surgery, Body mass index, Cross-sectional studies, Health surveys, Obesity, Public health surveillance

## Abstract

**Background:**

Updated recommendations for metabolic and bariatric surgery may expand the population requiring further clinical evaluation, but their scope across settings remains unclear. We estimated adults aged 18–69 years meeting a restricted surveillance-based operationalization of the 1991 US National Institutes of Health (NIH) and 2022 American Society for Metabolic and Bariatric Surgery/International Federation for the Surgery of Obesity and Metabolic Disorders (ASMBS/IFSO) thresholds through 2030.

**Methods:**

This was an observational burden-estimation and population-projection study based on secondary analysis of cross-sectional surveys. We analyzed World Health Organization (WHO) STEPwise Approach to Noncommunicable Disease Risk Factor Surveillance (STEPS) surveys from 37 settings. Estimates incorporated the complex survey design, demographic projections, paired primary-sampling-unit bootstrap resampling, and Shapley decomposition. The 2030 demographic-stability scenario was primary; the epidemiological-trend scenario was exploratory.

**Results:**

We analyzed 138,695 participants, representing 468.2 million adults in 2030. In the common classifiable universe, 17.1 million (3.6%) met the operationalized NIH threshold and 48.5 million (10.4%) met the ASMBS/IFSO threshold, a paired ratio of 2.84. The additional 31.5 million comprised 26.5 million with body mass index (BMI) 30.0–34.9 kg/m^2^ and metabolic disease and 4.9 million with BMI 35.0–39.9 kg/m^2^ outside the NIH threshold stratum. Threshold prevalence was higher among women and older adults and varied across settings. In the high/exploratory scenario, 64.2 million met the ASMBS/IFSO threshold, and epidemiological change accounted for 51.7% of the projected increase.

**Conclusion:**

The restricted surveillance-based operationalization of the ASMBS/IFSO thresholds identified a population nearly three times larger than the NIH threshold stratum. These estimates quantify a population requiring further clinical evaluation and capacity planning; they do not establish individual candidacy, indication, or expected utilization.

## Highlights

• Thirty-seven WHO STEPS settings represented 468.2 million adults in 2030.
• ASMBS/IFSO thresholds identified 48.5 million potentially eligible adults.
• Eligibility was 2.86 times higher than under the 1991 NIH criteria.
• Most newly eligible adults had BMI 30.0–34.9 kg/m^2^ and metabolic disease.
• Expanded eligibility requires evaluation capacity, equity, and follow-up.

## Graphical abstract

**Figure:**
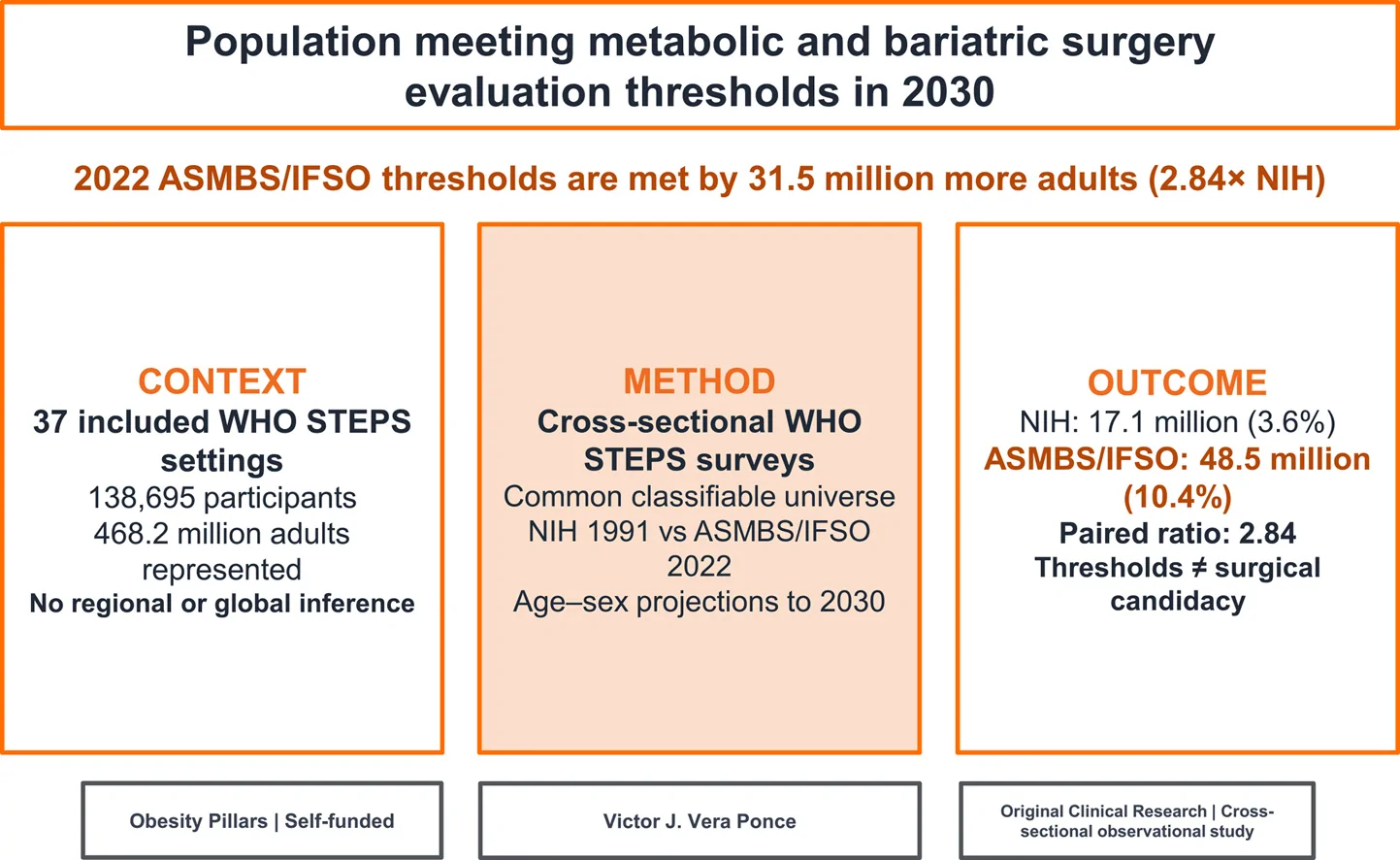

## 1 Introduction

Rising obesity has become one of the major public health challenges. In 2022, more than 1 billion people worldwide were living with obesity, following sustained increases among women and men since 1990 [1]. If observed trends continue, by 2050 approximately 1.95 billion adults aged 25 years or older would be living with obesity and 3.80 billion would have overweight or obesity [2]. The largest increases are expected in regions that are also undergoing rapid population growth and aging. Thus, epidemiologic and demographic change may simultaneously increase the need for interventions for obesity and its metabolic complications.

Metabolic and bariatric surgery is an option for people with clinically severe obesity or associated metabolic disease. A synthesis of controlled studies and matched cohorts found lower all-cause mortality and longer life expectancy with surgery than with nonsurgical management, particularly among people with diabetes [3]. However, the population requiring further evaluation depends on the threshold applied. The 1991 National Institutes of Health (NIH) criteria focused on high body mass index (BMI) thresholds and comorbidity [4]. By contrast, the 2022 recommendations of the American Society for Metabolic and Bariatric Surgery and the International Federation for the Surgery of Obesity and Metabolic Disorders (ASMBS/IFSO) included all people with BMI ≥35 kg/m^2^ and selected people with BMI 30.0–34.9 kg/m^2^ and metabolic disease [5]. Quantifying the change in population-level threshold attainment does not imply that meeting a threshold is equivalent to receiving an individual indication, accessing care, or undergoing a procedure.

This issue is particularly important in Latin America and the Caribbean. Between 1990 and 2021, obesity increased substantially in the region, and projections indicate that this burden will continue to grow [2]. Although metabolic and bariatric surgery is performed in several Latin American countries, access remains limited by cost, service availability, and shortages of specialized capacity [6]. In Peru, an analysis of national surveys from 2014 to 2022 found an increase in adults meeting population criteria for pharmacologic treatment or bariatric-surgery evaluation; that analysis applied only the 1991 NIH thresholds [7]. Updated and comparable estimates are therefore needed for service planning.

Evidence on population-level attainment of bariatric-surgery evaluation thresholds remains limited. Available studies have focused on individual countries, including the United States, England, and Peru, and most used national criteria or the 1991 NIH thresholds [[7], [8], [9]]. To our knowledge, no comparable multinational analysis has quantified the expansion produced by a harmonized surveillance-based operationalization of the 2022 ASMBS/IFSO thresholds, described differences across included settings, sex, and age, and projected the population meeting those thresholds while accounting for demographic structure and obesity trends. This information gap hampers anticipation of evaluation needs, surgical infrastructure, and long-term follow-up.

Therefore, the objective of this study was to estimate the prevalence and number of adults aged 18–69 years meeting restricted surveillance-based operationalizations of the 1991 NIH and 2022 ASMBS/IFSO population thresholds across 37 national and territorial settings and to project these estimates to 2030. We also evaluated the mutually exclusive components of the expansion, descriptive differences across included settings grouped by WHO region, sex and age, the separate contribution of demographic and epidemiological change, and robustness to alternative analytic decisions. The estimates are intended to inform planning of evaluation capacity without replacing individual clinical assessment or being interpreted as expected utilization.

## 2 Methods

### 2.1 Study design

This was an observational burden-estimation and population-projection study based on secondary analysis of cross-sectional surveys. The unit of analysis was the adult participant, and the projection unit was each national or territorial setting with an eligible World Health Organization (WHO) STEPwise Approach to Noncommunicable Disease Risk Factor Surveillance (STEPS) survey round. Reporting followed the Strengthening the Reporting of Observational Studies in Epidemiology (STROBE) statement and the Guidelines for Accurate and Transparent Health Estimates Reporting (GATHER) [10,11]. The STROBE checklist is presented in Table S1.

### 2.2 Data sources and survey selection

We used anonymized STEPS microdata accessed through the WHO Noncommunicable Disease (NCD) Microdata Repository under the access and use conditions applicable to each dataset [12]. STEPS collects information through interviews (Step 1), physical measurements (Step 2), and, when available, biochemical measurements (Step 3) using standardized instruments [13]. Rounds conducted between 2015 and 2024 were audited. Original files were retained in read-only mode, and their path, size, date, and Secure Hash Algorithm 256-bit (SHA-256) hash were recorded before transformation. Restricted microdata were not redistributed.

For the main analysis, one round per setting was selected. The survey had to represent the same national or territorial denominator used in the projection, be identified as the primary round, cover ages 18–69 years, allow formation of the eight prespecified sex-by-age cells, and include a useable Step 2 wt, primary sampling unit (PSU), and stratum. Repeated nonprimary rounds were reserved for temporal validation or sensitivity analyses, and subnational surveys were not combined with national projections. The complete inventory and inclusion decisions are reported in Table S3.

### 2.3 Population and analytic sample

The analytic population included participants aged 18–69 years whose sex could be harmonized as male or female, with valid height and weight, a positive Step 2 sampling weight, and complete PSU and stratum information. Women with known pregnancy were excluded because BMI does not comparably represent adiposity during gestation; unknown pregnancy was not treated as confirmed. Age groups were prespecified as 18–29, 30–44, 45–59, and 60–69 years. Individual filters were applied to 46 candidate setting-year files before setting-level eligibility gates; the reconstructed flow is presented in Fig. S1 and Table S4.

### 2.4 Harmonization and quality control

A common dictionary was constructed for age, sex, pregnancy, height, weight, blood pressure, diagnosis and treatment of hypertension and diabetes, sampling weight, cluster, and stratum. Each source variable was selected using its label, code, and storage type, not only the column name. Heights recorded in meters were converted to centimeters, and values between 100 and 250 cm were accepted. For weight, values between 25 and 300 kg were accepted, and conversion from grams was performed when applicable. BMI was calculated as weight in kilograms divided by height in meters squared, and values between 10 and 80 kg/m^2^ were retained.

For blood pressure, systolic values between 60 and 260 mm Hg and diastolic values between 30 and 180 mm Hg were accepted. Measured blood pressure was calculated as the mean of the last two valid readings, provided that at least two measurements were available. Before obtaining the results, unit tests with synthetic records at the limits of each criterion, denominator audits, and checks of age-by-sex cell coverage were performed. All derived variables retained the source file and row to ensure traceability.

### 2.5 Variables and operational definitions

Hypertension was defined as mean systolic blood pressure ≥140 mm Hg, mean diastolic blood pressure ≥90 mm Hg, reported use of antihypertensive medication, or prior diagnosis. In the main analysis, diabetes was defined by prior diagnosis or reported use of glucose-lowering medication. Metabolic disease was defined as hypertension or diabetes because these were the only weight-related conditions consistently harmonizable across all rounds. This restricted definition does not represent every condition that may influence clinical consideration of metabolic and bariatric surgery.

Under the restricted surveillance-based operationalization used here, adults met the 1991 NIH population threshold if BMI was ≥40 kg/m^2^ or 35.0–39.9 kg/m^2^ with observed metabolic disease. Adults met the 2022 ASMBS/IFSO population threshold if BMI was ≥35 kg/m^2^ or 30.0–34.9 kg/m^2^ with observed metabolic disease. For paired comparisons and the component decomposition, both thresholds were estimated in the common universe classifiable under ASMBS/IFSO. Adults with BMI 30.0–34.9 kg/m^2^ and missing metabolic-disease information are determinately negative for NIH but indeterminate for ASMBS/IFSO; the previous criterion-specific NIH support was therefore retained only for audit, whereas Table 2 uses the common universe. The operationalization does not capture previous treatment response, all obesity-related conditions, contraindications, operative risk, multidisciplinary assessment, or patient preference and consequently does not establish complete clinical eligibility or surgical candidacy. Full definitions and the denominator reconciliation are provided in Tables S2 and S7.

### 2.6 Demographic data and projection scenarios

Denominators by single year of age, sex, setting, and year were obtained from World Population Prospects 2024 (WPP 2024) of the United Nations Population Division [14]. Ages 18–69 years were grouped into the four prespecified intervals and matched to the same setting universe represented by each survey. Coverage of WHO regions was calculated only to quantify external validity; because settings were included by data availability and analytic eligibility rather than sampled from WHO regions, grouped estimates represent included settings only and are not regional or global estimates (Tables 1, S3, and S8; Fig. S2).

**Table 1:** Data coverage and analytic characteristics of included settings, grouped by World Health Organization region.

WHO region	Candidate surveys	Included settings	Median survey year	Analytic participants	Included 2030 population, ages 18–69	Coverage, %
Africa	10	9	2017	42,341	167,932,742	21.7
Americas	7	7	2019	22,618	23,308,875	3.2
Eastern Mediterranean	7	5	2017	24,497	120,690,804	22.1
Europe	9	9	2017	32,119	82,379,160	13.3
South-East Asia	0	0	–	0	0	0.0
Western Pacific	13	7	2015	17,120	73,864,168	4.8
Total candidate surveys/included settings	46	37	2017	138,695	468,175,749	Not applicable

Note. Candidate surveys are setting-year files. Regional grouping is descriptive; the included settings were not sampled to represent WHO regions. Coverage refers to the proportion of the 2030 WHO-region population aged 18–69 years represented by included settings. Abbreviations: WHO, World Health Organization; STEPS, STEPwise Approach to Noncommunicable Disease Risk Factor Surveillance.

**Table 2:** Paired estimates of adults meeting operationalized NIH 1991 and ASMBS/IFSO 2022 population thresholds in the common classifiable universe.

Scenario	Threshold or contrast	Adults (95% limits)	Prevalence or difference, % (95% limits)	Paired ratio (95% limits)	Interval type
Survey-year baseline	NIH 1991 threshold stratum	13,358,000 (12,778,000 to 14,022,000)	3.6 (3.5–3.8)	–	Design-based 95% CI
Survey-year baseline	ASMBS/IFSO 2022 threshold	38,127,000 (37,058,000 to 39,240,000)	10.4 (10.1–10.7)	–	Design-based 95% CI
Survey-year baseline	Increment beyond NIH threshold stratum	24,770,000 (23,914,000 to 25,671,000)	6.7 (6.5–7.0)	2.85 (2.75–2.96)	Design-based 95% CI
2030 demographic stability (primary)	NIH 1991 threshold stratum	17,070,000 (16,358,000 to 17,892,000)	3.6 (3.5–3.8)	–	Design-based 95% CI
2030 demographic stability (primary)	ASMBS/IFSO 2022 threshold	48,549,000 (47,275,000 to 49,849,000)	10.4 (10.1–10.6)	–	Design-based 95% CI
2030 demographic stability (primary)	Increment beyond NIH threshold stratum	31,478,000 (30,521,000 to 32,518,000)	6.7 (6.5–6.9)	2.84 (2.74–2.95)	Design-based 95% CI
2030 epidemiological trend (high/exploratory)	NIH 1991 threshold stratum	23,415,000 (22,285,000 to 24,599,000)	5.0 (4.8–5.3)	–	Conditional 95% UI
2030 epidemiological trend (high/exploratory)	ASMBS/IFSO 2022 threshold	64,153,000 (62,448,000 to 65,944,000)	13.7 (13.3–14.1)	–	Conditional 95% UI
2030 epidemiological trend (high/exploratory)	Increment beyond NIH threshold stratum	40,738,000 (39,537,000 to 42,004,000)	8.7 (8.4–9.0)	2.74 (2.64–2.84)	Conditional 95% UI

Note. Counts are rounded to the nearest 1000; unrounded values were used for calculations. Survey-year and 2030 demographic-stability limits are 95% design-based bootstrap percentile confidence intervals. High/exploratory epidemiological-trend limits are 95% conditional bootstrap percentile uncertainty intervals and do not represent complete uncertainty for unobserved settings, WHO regions, or the world. Abbreviations: ASMBS, American Society for Metabolic and Bariatric Surgery; BMI, body mass index; CI, confidence interval; IFSO, International Federation for the Surgery of Obesity and Metabolic Disorders; NIH, National Institutes of Health; UI, uncertainty interval.

In the primary scenario, sex- and age-specific prevalences observed in STEPS were held constant through 2030, so projected change depended only on population size and composition. A high/exploratory epidemiological scenario additionally incorporated sex-specific trends in age-standardized obesity prevalence from the WHO Global Health Observatory (GHO) [15]. Candidate trend windows and attenuation factors were governed by internal rolling-origin assessment and direct repeated-STEPS validation; because the epidemiological specification was less accurate in the directly testable comparison, demographic stability remained primary.

The target obesity prevalence for 2030 was calculated by adding the selected trend to the baseline logit. Then, within each setting-by-sex combination, a constant shift was added to individual BMI until the weighted target prevalence of BMI ≥30 kg/m^2^ was reproduced. This procedure preserved the rank order of the BMI distribution and kept metabolic disease fixed. An alert was established for absolute shifts greater than 3 kg/m^2^. This scenario was considered high and exploratory because it depends on trend stability, structural calibration, and assumptions that cannot be identified from a single round.

### 2.7 Statistical analysis

Each survey design incorporated the Step 2 wt, PSU, and stratum nested by setting. Single PSUs within a stratum were handled using a conservative lonely-PSU adjustment. Estimates were calculated within prespecified sex-by-age cells and projected with matched WPP denominators. Primary comparisons of NIH and ASMBS/IFSO thresholds were paired within the common classifiable universe, preserving covariance for differences and ratios. Descriptive aggregates by WHO region were calculated only across the included settings and were not interpreted as estimates for complete regions.

Uncertainty was propagated with 1000 paired bootstrap replicates generated by resampling PSUs within strata, using seed 20260724 [16]. For survey-year estimates and the 2030 demographic-stability projection, the 2.5th and 97.5th percentiles are reported as design-based 95% confidence intervals (CIs), conditional on the observed survey design and fixed WPP denominators. For the high/exploratory epidemiological-trend projection, the Shapley decomposition, and trend-dependent sensitivities, the corresponding percentiles are reported as 95% conditional uncertainty intervals (UIs). These UIs incorporate the propagated sampling and trend components implemented by the model but not complete uncertainty from setting selection, WPP/GHO inputs, model selection, or structural assumptions. The interval ledger is provided in Table S10.

Projected change was divided into population size, age-sex composition, and cell-specific prevalence using an exact Shapley decomposition [17]. Contributions are descriptive allocations across modeled components, not causal effects. Population estimates in the main tables were rounded to the nearest 1000 and narrative counts to one decimal million; unrounded values were retained for computation and exact closure.

### 2.8 Validation and sensitivity analyses

Temporal validity was evaluated in the only setting with two comparable rounds. The earlier round was used to project the later round, and the absolute relative error of the purely demographic scenario was compared with epidemiologic specifications that could be evaluated without using future information. The WHO trend window was selected separately through internal rolling-origin validation. Based on these results, the primary and exploratory scenarios were defined before drafting the findings.

Robustness analyses varied age range, small-cell handling, survey round, regional trend backup, demographic variant, trend window and attenuation, epidemiological scenario, and missing-classification assumptions. Following the 2022 ASMBS/IFSO recommendation for Asian populations, a BMI threshold of 27.5 kg/m^2^ was evaluated geographically because harmonizable individual-level ethnicity or ancestry was unavailable [5,18]. The narrower prespecified proxy applied the threshold to settings classified by the United Nations Standard Country or Area Codes for Statistical Use (M49) in Eastern, South-eastern, or Southern Asia [19]. A second boundary analysis applied it to all included settings geographically classified in Asia by M49, including Western and Central Asian settings; this broad analysis was explicitly exploratory. M49 is a statistical geographic classification, not an ethnic or clinical classification, and neither sensitivity establishes individual candidacy [18,19].

The contribution of Step 3 was evaluated only in rounds with fasting glucose or glycated hemoglobin, a Step 3 wt, and a complete design. In the same participants, diabetes was updated using fasting glucose ≥126 mg/dL (7.0 mmol/L), glycated hemoglobin ≥6.5% (48 mmol/mol), prior diagnosis, or reported treatment [20]. Paired comparisons used the Step 3 wt and 1000 cluster replicates. Missing data for classification were evaluated using two extreme bounds, classifying all indeterminate cases as ineligible or eligible, and using inverse-probability weighting among classifiable records.

### 2.9 Software and reproducibility

Data management, complex-survey estimation, projections, resampling, and validation were conducted in R 4.6.1.

### 2.10 Ethical considerations

This secondary analysis used anonymized microdata accessed under repository-specific conditions and involved no participant contact, intervention, or access to direct identifiers [21]. The authors did not seek additional institutional review or new informed consent for this analysis. No formal waiver or exemption document was available, and therefore no confirming document number is reported. The original surveys were conducted under the responsibility of national teams in accordance with local ethical and consent requirements, and the present analysis complied with repository access and use conditions. No attempt was made to reidentify any participant.

## 3 Results

### 3.1 Sample selection and characteristics

We identified 46 candidate setting-year files containing 174,542 records. After restricting to ages 18–69 years, known binary sex, valid measured anthropometry, a positive Step 2 wt, and complete survey-design identifiers, 154,265 records remained. Exclusion of 57 known pregnancies yielded 154,208 individual analytic records across all candidate settings; subsequent setting-level eligibility gates produced 138,695 participants in 37 included settings. The identity 154,265 − 57 = 154,208 and the distinction between retained counts and exclusions were independently validated (Fig. S1 and Table S4).

The 37 included settings comprised nine in Africa, seven in the Americas, five in the Eastern Mediterranean, nine in Europe, and seven in the Western Pacific; no setting from South-East Asia was included. Together they represented 468,175,749 adults aged 18–69 years in 2030. Coverage of the corresponding WHO-region populations was 21.7% in Africa, 3.2% in the Americas, 22.1% in the Eastern Mediterranean, 13.3% in Europe, 0% in South-East Asia, and 4.8% in the Western Pacific. These are partial, non-probability setting coverages and do not support regional or global inference (Table 1; Tables S3 and S8; Fig. S2).

### 3.2 Eligibility projections to 2030

In the survey-year population, 13.4 million adults (3.6%; 95% design-based CI, 3.5%–3.8%) met the operationalized NIH threshold and 38.1 million (10.4%; 10.1%–10.7%) met the ASMBS/IFSO threshold in the common classifiable universe; the paired ratio was 2.85 (2.75–2.96). In the primary 2030 demographic-stability scenario, the corresponding estimates were 17.1 million (3.6%; 3.5%–3.8%) and 48.5 million (10.4%; 10.1%–10.6%), with an increment of 31.5 million and a paired ratio of 2.84 (2.74–2.95). In the high/exploratory scenario, 23.4 million (5.0%; 95% conditional UI, 4.8%–5.3%) met the NIH threshold and 64.2 million (13.7%; 13.3%–14.1%) met the ASMBS/IFSO threshold; the paired ratio was 2.74 (2.64–2.84) (Table 2 and Table S5).

Among the included settings grouped descriptively by WHO region, the 2030 primary ASMBS/IFSO threshold prevalence was 19.3% in the European group, 16.0% in the Eastern Mediterranean group, 16.0% in the Americas group, 4.9% in the African group, and 1.7% in the Western Pacific group. South-East Asia had no included setting. These aggregates are not region-wide estimates; detailed setting-level results are provided in Table S13, Supplementary Dataset S1, and Fig. S3.

### 3.3 Components and between-setting heterogeneity

**Fig. 1:**
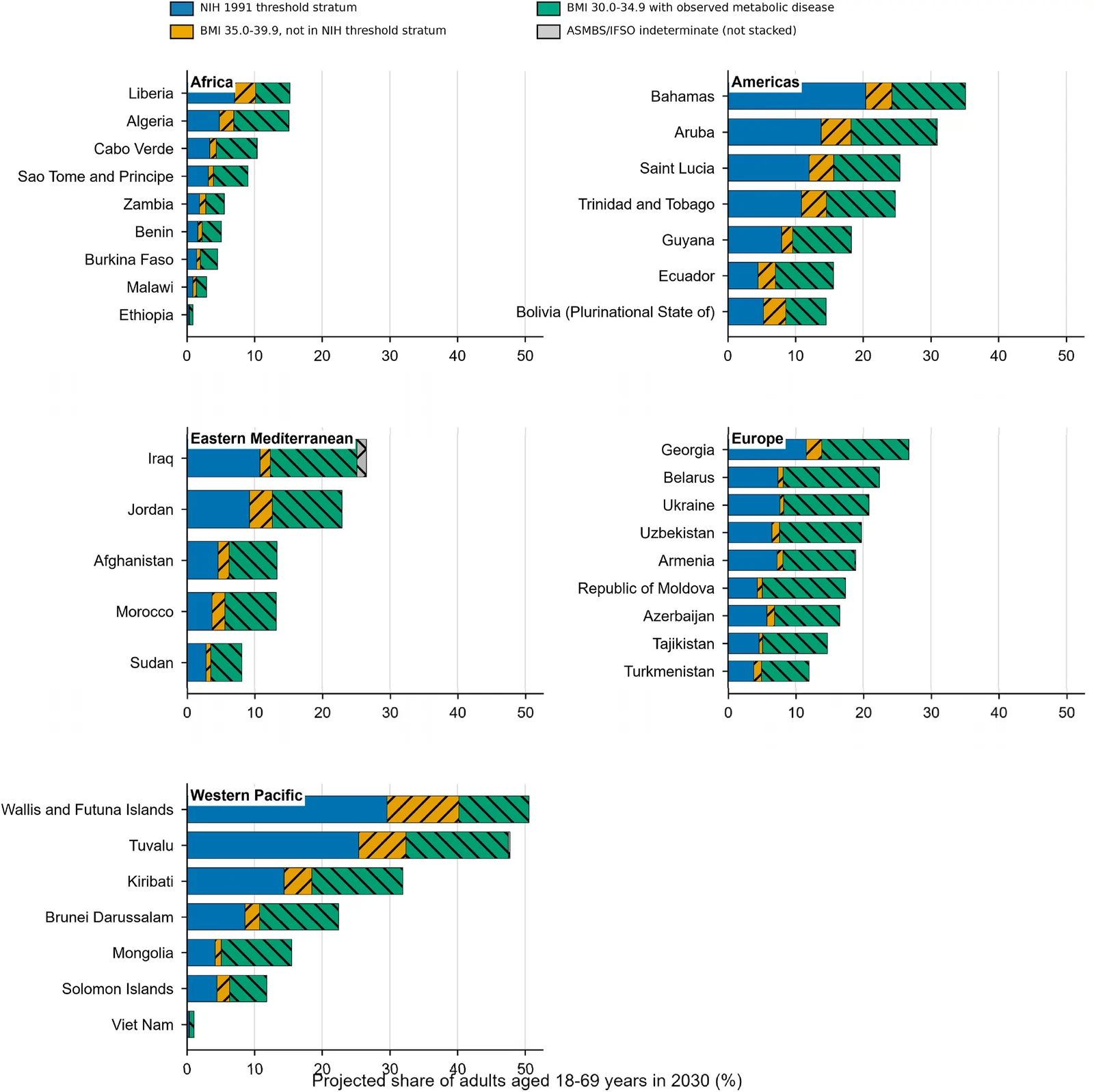
Mutually exclusive components of adults meeting the 2030 operationalized ASMBS/IFSO population threshold under demographic stability. Note. The NIH threshold stratum, BMI 35.0–39.9 kg/m^2^ outside the NIH threshold stratum, and BMI 30.0–34.9 kg/m^2^ with observed metabolic disease sum exactly to the ASMBS/IFSO total. ASMBS/IFSO-indeterminate status is displayed separately and is not stacked as threshold attainment. Abbreviations: ASMBS, American Society for Metabolic and Bariatric Surgery; BMI, body mass index; IFSO, International Federation for the Surgery of Obesity and Metabolic Disorders; NIH, National Institutes of Health.

Of the 48.5 million adults meeting the operationalized ASMBS/IFSO threshold in the primary 2030 scenario, 17.1 million were in the NIH threshold stratum, 26.5 million had BMI 30.0–34.9 kg/m^2^ with observed metabolic disease, and 4.9 million had BMI 35.0–39.9 kg/m^2^ outside the NIH threshold stratum. The three components were mutually exclusive and summed exactly to the ASMBS/IFSO total; indeterminate status was reported separately. The NIH stratum is identical to the revised common-universe NIH total in Table 2 (Fig. 1; Tables S6 and S7).

**Fig. 2:**
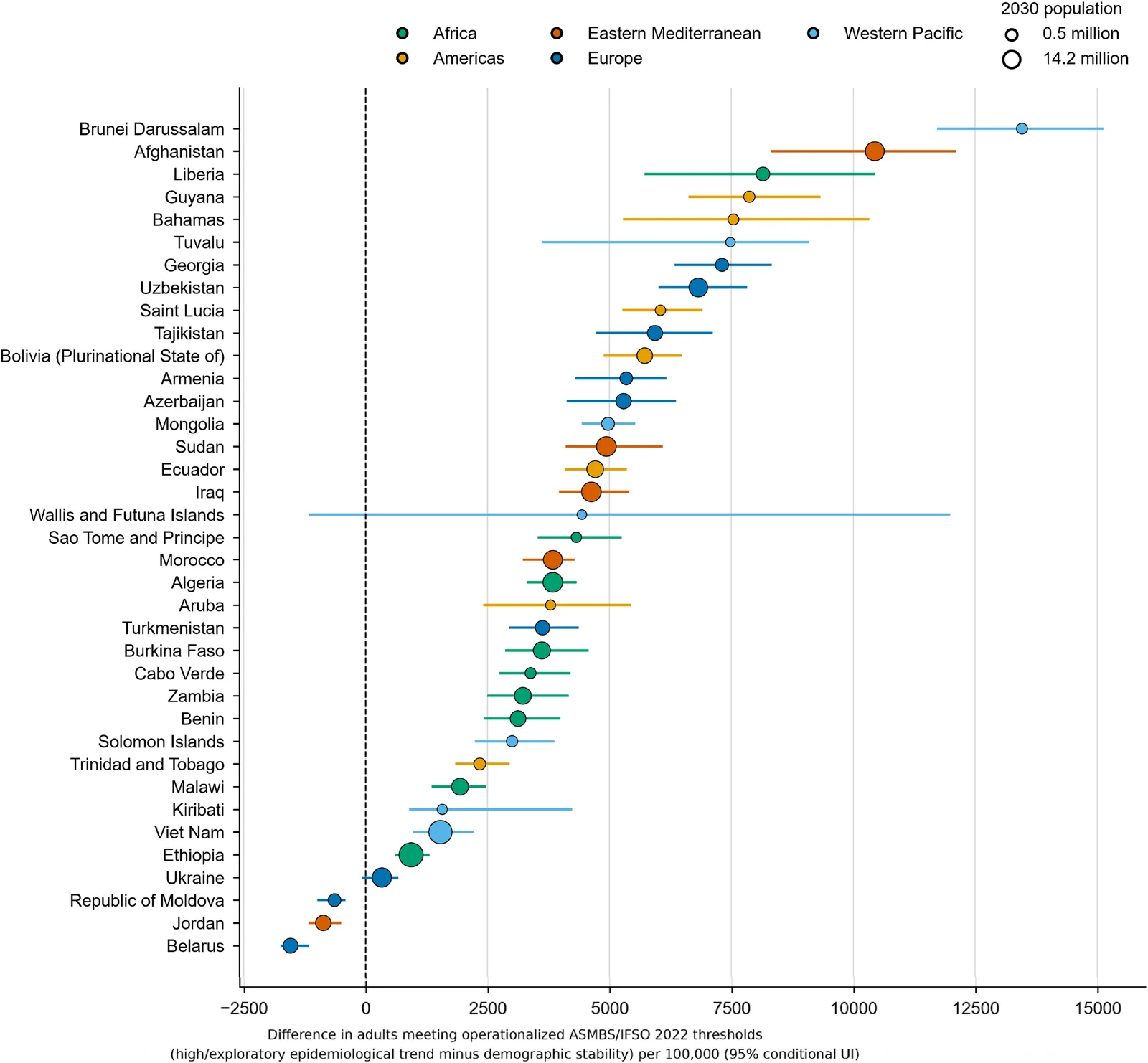
Setting-level difference between the high/exploratory epidemiological-trend scenario and demographic stability. Note. The x-axis is a scenario difference per 100,000 adults, not an absolute burden or causal effect. Horizontal intervals are 95% conditional uncertainty intervals. Abbreviations: ASMBS, American Society for Metabolic and Bariatric Surgery; IFSO, International Federation for the Surgery of Obesity and Metabolic Disorders; UI, uncertainty interval.

The high/exploratory scenario produced a higher estimate than demographic stability in 34 settings and a lower estimate in three. The largest positive difference was observed in Brunei Darussalam, with 13,460 additional adults per 100,000 (95% conditional UI, 11,716–15,131), followed by Afghanistan with 10,441 per 100,000. Negative differences occurred in Belarus, Jordan, and the Republic of Moldova. These are scenario contrasts, not causal effects (Fig. 2).

### 3.4 Distribution by age and sex

**Fig. 3:**
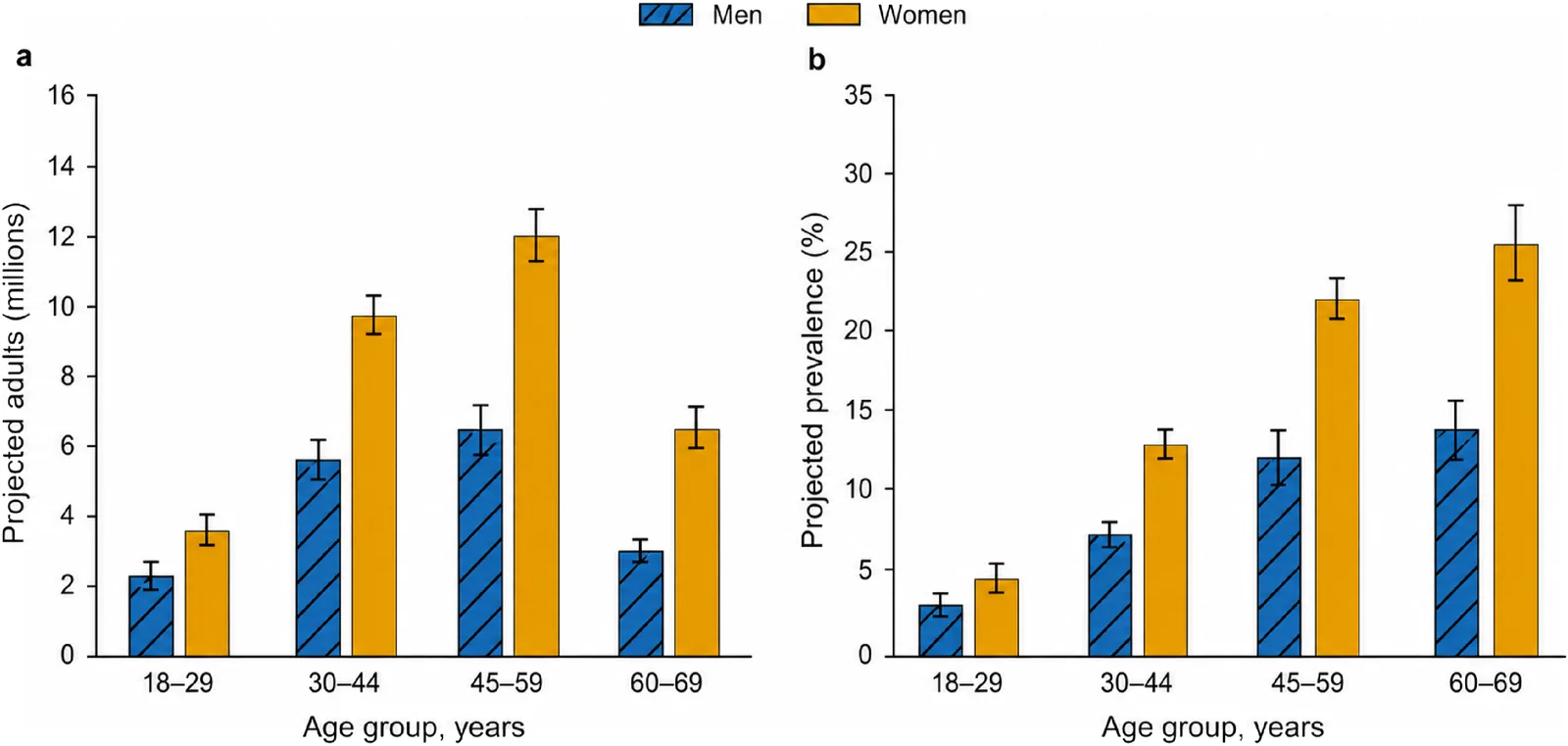
Projected 2030 counts and prevalence by age and sex under the demographic-stability primary scenario. Note. Panel a presents projected counts and panel b presents prevalence. Whiskers are paired 95% design-based bootstrap percentile confidence intervals. Abbreviation: CI, confidence interval.

In the primary demographic-stability scenario, the prevalence of meeting the operationalized ASMBS/IFSO threshold was higher among women in every age group. Among women, it increased from 4.3% (95% design-based CI, 3.8%–4.9%) at ages 18–29 years to 25.2% (24.0%–26.5%) at ages 60–69 years. Among men, it increased from 2.8% (2.3%–3.3%) at ages 18–29 years to 13.7% (12.7%–14.7%) at ages 60–69 years. These descriptive strata were not formal interaction tests (Fig. 3; Table S14).

### 3.5 Decomposition of projected change

Between the survey year and 2030, the high/exploratory scenario projected an increase of 26.0 million adults meeting the operationalized ASMBS/IFSO threshold. Exact Shapley decomposition attributed 13.5 million (95% conditional UI, 12.7–14.2 million; 51.7%) to epidemiological change, 10.0 million (9.6–10.4 million; 38.4%) to population size, and 2.6 million (2.4–2.7 million; 9.9%) to age-sex composition. These contributions are descriptive allocations within the modeled scenario and are not causal effects (Table 3).

**Table 3:** Exact Shapley decomposition of change in adults meeting the operationalized ASMBS/IFSO 2022 population threshold under the high/exploratory scenario.

	Total high-trend change in adults	Shapley contribution (95% conditional UI)	Share of total change, %
Panel A. All included settings - Population size	26,025,881	9,994,994 (9,597,953 to 10,423,005)	38.4
Panel A. All included settings - Age-sex composition	26,025,881	2,565,814 (2,436,237 to 2,681,644)	9.9
Panel A. All included settings - Epidemiological change	26,025,881	13,465,073 (12,682,905 to 14,244,257)	51.7
Panel B. Included African settings - Population size	6,173,005	2,490,527 (2,317,882 to 2,687,942)	40.3
Panel B. Included African settings - Age-sex composition	6,173,005	535,212 (463,562 to 599,238)	8.7
Panel B. Included African settings - Epidemiological change	6,173,005	3,147,266 (2,824,514 to 3,488,458)	51.0
Panel C. Included settings in the Americas - Population size	1,887,257	654,998 (616,990 to 692,243)	34.7
Panel C. Included settings in the Americas - Age-sex composition	1,887,257	166,192 (147,938 to 185,188)	8.8
Panel C. Included settings in the Americas - Epidemiological change	1,887,257	1,066,067 (967,136 to 1,154,244)	56.5
Panel D. Included Eastern Mediterranean settings - Population size	13,036,924	6,714,902 (6,380,550 to 7,044,166)	51.5
Panel D. Included Eastern Mediterranean settings - Age-sex composition	13,036,924	900,718 (829,952 to 963,551)	6.9
Panel D. Included Eastern Mediterranean settings - Epidemiological change	13,036,924	5,421,304 (4,870,955 to 5,892,366)	41.6
Panel E. Included European settings - Population size	3,497,562	−51,850 (−183,302 to 91,189)	−1.5
Panel E. Included European settings - Age-sex composition	3,497,562	928,997 (859,719 to 994,337)	26.6
Panel E. Included European settings - Epidemiological change	3,497,562	2,620,415 (2,381,110 to 2,873,884)	74.9
Panel F. Included Western Pacific settings - Population size	1,431,133	186,417 (163,529 to 214,931)	13.0
Panel F. Included Western Pacific settings - Age-sex composition	1,431,133	34,695 (2565 to 74,353)	2.4
Panel F. Included Western Pacific settings - Epidemiological change	1,431,133	1,210,021 (829,883 to 1,675,257)	84.5

Note. Contributions sum exactly to the modeled full-trend change. Limits are 95% conditional bootstrap percentile uncertainty intervals; negative or greater-than-100% shares are retained when offsetting contributions occur. Regional panels represent only included settings grouped by WHO region. Abbreviations: ASMBS, American Society for Metabolic and Bariatric Surgery; IFSO, International Federation for the Surgery of Obesity and Metabolic Disorders; UI, uncertainty interval; WHO, World Health Organization.

### 3.6 Validation and sensitivity analyses

In temporal validation using repeated STEPS rounds, the demographic-only projection had an absolute relative error of 8.0%, whereas the best directly evaluable epidemiological specification had an error of 37.4%. The GHO window beginning in 2015 had the best internal rolling-origin performance but could not be directly evaluated using the repeated 2015-to-2021 STEPS contrast. Demographic stability was therefore retained as primary and the epidemiological-trend projection as high/exploratory (Table S12; Figs. S4 and S5).

Restricting the population to ages 20–69 years increased projected prevalence by 0.54 percentage points, although counts were not comparable because the population universe changed. Jeffreys smoothing on the same universe increased prevalence by 0.15 percentage points and the count by approximately 0.7 million. Replacing the 2021 Viet Nam round with the 2015 round changed prevalence by 0.02 percentage points and the count by approximately 0.1 million, without a causal pandemic interpretation. Regional trend backup changed the total by fewer than 0.01 million adults. In the paired Step 3 sample, adding fasting glucose or glycated hemoglobin increased prevalence by 0.32 percentage points and the count by approximately 0.8 million (Table S12 and Fig. S6).

Under the narrower M49 proxy limited to Eastern, South-eastern, or Southern Asia, applying BMI 27.5 kg/m^2^ in four settings yielded 57.0 million adults (12.2%), 8.4 million and 1.8 percentage points above the primary estimate. The broad all-M49 Asia boundary analysis applied the threshold in 12 settings and yielded 76.5 million adults (16.3%), 27.9 million and 6.0 percentage points above primary. The broad analysis includes Western and Central Asian settings, including Iraq, Jordan, Armenia, Azerbaijan, Georgia, Tajikistan, Turkmenistan, and Uzbekistan, solely because of M49 geography. Neither analysis identifies ethnicity or supports a setting-wide clinical recommendation or individual candidacy determination (Table S9 and Fig. S7).

In the leave-one-setting-out analysis, the prevalence difference from the primary estimate ranged from −1.15 percentage points when Iraq was excluded to 2.03 points when Ethiopia was excluded (Fig. S8). The maximum ASMBS/IFSO-indeterminate proportion was 4.48% in Iraq, and the smallest classifiable cell contained 40 participants in Kiribati (Fig. S9). All 12 reanalysis-specific analytic controls passed, including common-universe NIH reconciliation, integer component closure, Shapley additivity, complete six-region representativeness accounting, and explicit separation of M49 geography from ethnic or clinical inference (Table S11).

Classifying all indeterminate cases as not meeting the threshold decreased the estimate by 0.08 percentage points and approximately 0.4 million adults; classifying all as meeting it increased the estimate by 0.24 points and approximately 1.1 million adults. Inverse-probability weighting did not change the primary estimate. These bounds do not alter the central interpretation (Table S12).

## 4 Discussion

### 4.1 Main findings

Across 37 included settings representing 468.2 million adults aged 18–69 years in 2030, the restricted surveillance-based operationalization of the 2022 ASMBS/IFSO population threshold was met by 10.4% and 48.5 million adults in the primary demographic-stability scenario. In the same common classifiable universe, 3.6% and 17.1 million met the operationalized 1991 NIH threshold, producing a paired ratio of 2.84. More than half of the ASMBS/IFSO total consisted of adults with BMI 30.0–34.9 kg/m^2^ and observed metabolic disease, while approximately one tenth had BMI 35.0–39.9 kg/m^2^ outside the NIH threshold stratum. Threshold attainment varied across included settings and was higher among women and older age groups. The high/exploratory scenario reached 64.2 million adults, with epidemiological change accounting for 51.7% of the modeled increase.

### 4.2 Comparison with other studies

The 3.6% prevalence under the common-universe NIH threshold was lower than the 12.6% reported in the United States and the 5.4% estimated in England, but higher than the 2.5% observed in Peru [[7], [8], [9]]. A later English analysis estimated that 7.3% of the population was potentially eligible and that only 0.20% received metabolic surgery annually [22]. Direct numerical comparison remains limited by differences in period, age range, metabolic-disease definitions, clinical rules, and population structure. Our analysis extends this evidence by applying harmonized surveillance rules across multiple settings and comparing the 1991 and 2022 thresholds within the same classifiable universe.

The 2.84-fold paired ratio quantifies the magnitude of the threshold expansion under the restricted operationalization. The largest additional stratum comprised 26.5 million adults with BMI 30.0–34.9 kg/m^2^ and observed metabolic disease, corresponding to the recommendation to consider selected people with class I obesity and metabolic disease [5]. The systematic review supporting the updated recommendations found evidence for 11 of 13 indications and noted that access to metabolic and bariatric surgery remains low in most countries [23]. Our results do not test the effectiveness of the recommendations or identify individual candidates; they estimate the population requiring further assessment if comparable threshold components were incorporated into care pathways.

Descriptive aggregates differed among included settings grouped by WHO region, consistent with heterogeneous obesity patterns and access barriers [2,6,24]. However, the settings were selected according to survey availability and analytic eligibility rather than sampled to represent regions; coverage ranged from 0% to 22.1%. The grouped estimates must therefore not be interpreted as WHO-region prevalence or burden. They instead illustrate heterogeneity among the observed settings and the potential mismatch between evaluation demand and service readiness.

Higher threshold attainment among women and older adults is consistent with population studies from England and Peru [7,9,22]. The population that ultimately undergoes surgery is nevertheless determined by referral, multidisciplinary assessment, contraindications, operative risk, previous treatment, informed preference, financing, and access. The IFSO Global Registry reported that 77.1% of operated patients from 2015 to 2018 were women [25], and the IFSO worldwide survey recorded 598,834 surgical and endoluminal procedures in 2021 [26]. That utilization count cannot be compared directly with 48.5 million adults meeting surveillance-based thresholds.

### 4.3 Implications for public and international health

Health planning should distinguish adults meeting population thresholds for further evaluation, adults receiving an indication after multidisciplinary assessment, and adults ultimately accessing a procedure. Projected counts should be used to anticipate evaluation demand and capacity rather than as operative targets. The WHO framework for obesity prevention and management calls for accessible, affordable, sustainable services integrated from primary care through specialized treatment and long-term follow-up [27]. Updating thresholds may require multidisciplinary teams, referral mechanisms, safe surgical centers, nutritional and metabolic follow-up, information systems, and capacity to manage complications and revisional surgery.

Expansion of population thresholds may also increase inequities. Without financing, transparent prioritization, and territorial expansion of services, people with lower coverage, lower income, or remote residence may face a wider gap between threshold attainment and actual evaluation. Planning should therefore integrate equity, clinical benefit, risk, preference, and system capacity rather than converting population counts into entitlement or utilization targets.

The principal contribution of this study is the combination of standardized surveillance, an explicit restricted operationalization, paired common-universe comparison, demographic projection, and reproducible scenario analysis across diverse included settings. The Shapley decomposition indicates that epidemiological change could contribute more than population growth in the high scenario, but this is a model-based descriptive allocation. The primary demographic scenario remains the more defensible planning benchmark because it performed better in direct temporal validation.

## 5 Limitations

Several limitations should be considered. First, the 37 settings depended on availability and quality of STEPS microdata from 2015 to 2024 and were not sampled to represent WHO regions; coverage was partial or absent, so no regional or global prevalence should be inferred. Second, the analysis combined cross-sectional surveys with external demographic and obesity-trend inputs, and the high scenario remains conditional on selected windows, calibration, and structural assumptions. Third, diabetes in the main analysis relied on diagnosis or medication because biomarkers and Step 3 wt were not consistently available; the paired biomarker sensitivity was therefore limited to a subset. Fourth, BMI is an imperfect proxy for adiposity and risk, especially across populations [28]. Fifth, harmonized metabolic disease was restricted to hypertension or diabetes and omitted other weight-related conditions. Sixth, the surveillance data did not capture treatment history, multidisciplinary assessment, contraindications, operative risk, preferences, costs, installed capacity, or probability of utilization. Finally, the M49 sensitivity was geographic rather than ethnic. Consequently, meeting the modeled thresholds identifies a population for further clinical evaluation and is not equivalent to complete guideline eligibility, an individual surgical indication, or expected procedures. Design-based CIs and conditional UIs quantify only the uncertainty sources explicitly propagated and do not address unobserved settings or complete model uncertainty.

## 6 Conclusion

The restricted surveillance-based operationalization of the 2022 ASMBS/IFSO population threshold identified 48.5 million adults across the 37 included settings in 2030, nearly three times the 17.1 million in the corresponding common-universe NIH threshold stratum. Most of the difference involved adults with BMI 30.0–34.9 kg/m^2^ and observed metabolic disease. These estimates quantify a population potentially requiring further clinical evaluation and capacity planning; they do not establish surgical candidacy, predict utilization, or replace individual assessment of benefits, risks, contraindications, preferences, and follow-up capacity.

Health systems may use these estimates to plan multidisciplinary evaluation, safe surgical capacity, equitable access, and long-term follow-up. Repeated national surveys and capacity audits should update the projections, while individual decisions remain based on clinical benefit, risk, informed preference, and follow-up capacity.

• In 2030, 10.4% (48.5 million) met the operationalized ASMBS/IFSO threshold versus 3.6% (17.1 million) in the common-universe NIH threshold stratum.
• Most of the additional population had BMI 30.0–34.9 kg/m^2^ with observed metabolic disease; meeting a surveillance threshold indicates need for assessment, not an individual surgical recommendation.
• Expanded population thresholds require multidisciplinary evaluation, surgical capacity, equitable access, and long-term follow-up.

## Ethical review

This secondary analysis used anonymized WHO STEPS microdata accessed under dataset-specific repository conditions. It involved no participant contact, intervention, or access to direct identifiers. The authors did not seek additional institutional review or new informed consent, and no formal waiver or exemption document was available; therefore, no protocol or waiver number is reported. Original surveys were conducted under the responsibility of national teams and applicable local ethical and consent requirements.

## Data availability

The underlying WHO STEPS microdata were accessed under dataset-specific repository conditions and cannot be redistributed by the author. Researchers must request access through the WHO NCD Microdata Repository.

## Author contributions

Victor J. Vera Ponce: Conceptualization, Investigation, Formal analysis, Methodology, Software, Funding acquisition, Supervision, Resources, Visualization, Project administration, Validation, Data curation, Writing – original draft, and Writing – review and editing.

## Statement on the use of artificial intelligence in writing

Generative artificial intelligence (AI) and large language models (LLMs) were not used in study conception, statistical analysis, or generation of results.

## Funding

The costs of processing the article, if applicable at the time of acceptance, will be covered by the funding source (Vice-Rectorate for Research of the National University Toribio Rodríguez de Mendoza of Amazonas), which had no role in the design of the study, the collection and analysis of data, the decision to publish or the preparation of the manuscript.

## Declaration of competing interests

The author declares no competing interests.
