# Supplementary material for: Population meeting surveillance-based thresholds for metabolic and bariatric surgery evaluation under the 1991 NIH and 2022 ASMBS/IFSO criteria across 37 settings: STEPS analysis and projections to 2030
Source: Obes Pillars. 2026 Aug 29;20:100329. doi: 10.1016/j.obpill.2026.100329 (PMC13562079; doi:10.1016/j.obpill.2026.100329)
Supplement: Supplementary file 1 [file mmc1.docx]

**Population meeting surveillance-based thresholds for metabolic and bariatric surgery evaluation under the 1991 NIH and 2022 ASMBS/IFSO criteria across 37 settings: STEPS analysis and projections to 2030**

Victor J. Vera Ponce

**Supplementary material**

This revised supplement reports the final common-universe comparison, regional-coverage accounting, M49 geographic sensitivity, interval classification, and reproducibility checks. The term “meeting an operationalized population threshold” does not denote individual eligibility, candidacy, indication, or expected use.

**Table S1. STROBE checklist for observational studies.**

| **STROBE section** | **Item** | **Recommendation** | **Compliance and stable location** |
| --- | --- | --- | --- |
| Title and abstract | 1a | Identify the study design using a commonly used term in the title or abstract. | Addressed. The title describes the STEPS analysis and projections, and Abstract—Methods begins with the complete study-design statement. |
| Title and abstract | 1b | Provide in the abstract an informative and balanced summary of what was done and the main findings. | Addressed. The abstract contains only Background, Methods, Results, and Conclusion and reports the common-universe estimates and interpretive restrictions. |
| Introduction | 2 | Explain the scientific background and rationale for the investigation. | Addressed in Introduction, which distinguishes population-threshold attainment from clinical candidacy and utilization. |
| Introduction | 3 | State specific objectives and, when applicable, prespecified hypotheses. | Addressed in the final Introduction paragraph with objectives for the NIH/ASMBS/IFSO comparison, projections, components, descriptive heterogeneity, and robustness. |
| Methods | 4 | Present key elements of the study design early in the paper. | Addressed at the start of Methods—Study design using a standard observational burden-estimation and population-projection formulation. |
| Methods | 5 | Describe the setting, locations, and relevant dates, including periods of recruitment, exposure, follow-up, and data collection. | Addressed in Methods—Data sources and survey selection and Table S3, including 37 settings, 2015–2024 rounds, ages 18–69 years, and projection through 2030. |
| Methods | 6a | Specify eligibility criteria and the sources and methods of participant selection. | Addressed in Methods—Population and analytic sample, Figure S1, and Tables S3–S4. |
| Methods | 6b | For matched studies, describe matching criteria and the number of exposed and unexposed participants. | Not applicable; participants were not matched. |
| Methods | 7 | Clearly define all outcomes, exposures, predictors, potential confounders, and effect modifiers, including diagnostic criteria. | Addressed in Methods—Variables and operational definitions and Table S2, including the restricted metabolic-disease definition and common classifiable universe. |
| Methods | 8 | For each variable, indicate the data source and measurement method, and explain comparability when more than one group is involved. | Addressed in Methods—Harmonization and quality control, Table S3, and the reproducibility package manifests. |
| Methods | 9 | Describe efforts to address potential sources of bias. | Addressed through harmonized rules, design-based estimation, a primary demographic scenario, sensitivity analyses, and explicit restriction of regional and clinical inference. |
| Methods | 10 | Explain how the study size was determined. | No formal power calculation was performed; all eligible records from selected survey rounds were used. |
| Methods | 11 | Explain how quantitative variables were handled and justify the groupings used. | Addressed for BMI, blood pressure, age groups, thresholds, and projected counts in Methods and Table S2. |
| Methods | 12a | Describe all statistical methods, including those used to control for confounding when applicable. | Addressed in Methods—Statistical analysis, including complex-survey design, paired common-universe comparison, projection, bootstrap, and Shapley decomposition. |
| Methods | 12b | Describe any methods used to examine subgroups and interactions. | Addressed for descriptive strata by included setting, WHO grouping, sex, and age; no formal interaction testing was performed. |
| Methods | 12c | Explain how missing data were addressed. | Addressed by explicit indeterminate classification and extreme-bound and inverse-probability-weighting sensitivities. |
| Methods | 12d | When applicable, describe analytical methods that account for the sampling strategy. | Addressed through setting-nested sampling weights, strata, PSUs, lonely-PSU handling, and PSU bootstrap resampling. |
| Methods | 12e | Describe any sensitivity analyses. | Addressed in Methods—Validation and sensitivity analyses and Tables S9 and S12. |
| Results | 13a | Report the numbers of individuals at each stage of the study. | Addressed in Results, Figure S1, and Table S4; retained counts and exclusions are reported separately. |
| Results | 13b | Give reasons for nonparticipation or exclusion at each stage. | Addressed in Table S3 and the reconstructed flow. |
| Results | 13c | Consider use of a flow diagram. | Addressed by Figure S1. |
| Results | 14a | Describe participant characteristics and relevant information on exposures and potential confounders. | Addressed by descriptive age-sex and setting-level outputs in Tables S13–S14. |
| Results | 14b | Indicate the number of participants with missing data for each variable of interest. | Addressed by classifiability, indeterminate-status, biomarker, and missing-classification analyses. |
| Results | 14c | For longitudinal studies, summarize follow-up time. | Not applicable; there was no individual longitudinal follow-up. |
| Results | 15 | Report numbers of outcome events or summary measures. | Addressed through prevalences, counts, paired differences, ratios, components, and scenario results in the main and supplementary tables. |
| Results | 16a | Provide unadjusted estimates and, when applicable, adjusted estimates with precision, making clear which variables were adjusted for and why. | Design-weighted estimates are reported. No causal or confounder-adjusted effect was estimated. |
| Results | 16b | Report category boundaries when continuous variables are categorized. | Addressed by reporting all prespecified threshold bands and age groups. |
| Results | 16c | When relevant, translate estimates of relative risk into absolute risk for a meaningful time period. | Addressed by presenting absolute projected counts together with prevalence. |
| Results | 17 | Report other analyses, such as subgroups, interactions, and sensitivity analyses. | Addressed in validation, sensitivity, regional-coverage, Asian-geographic, and QA outputs. |
| Discussion | 18 | Summarize key results with reference to study objectives. | Addressed in Discussion—Main findings. |
| Discussion | 19 | Discuss limitations, including potential sources of bias or imprecision and, when possible, their direction and magnitude. | Addressed in one consolidated Limitations paragraph, including incomplete setting coverage, data harmonization, modeling assumptions, missing clinical dimensions, M49 geography, and interval scope. |
| Discussion | 20 | Give a cautious overall interpretation considering objectives, limitations, multiplicity of analyses, and external evidence. | Addressed with cautious, noncausal interpretation and comparison with prior studies. |
| Discussion | 21 | Discuss the generalizability or external validity of the results. | Addressed. Findings are explicitly limited to included settings; no WHO-region or global inference is made. |
| Other information | 22 | Give the source of funding and the role of the funders for the present study and, when applicable, for the original studies. | Addressed in the Funding declaration. |

Note. STROBE, Strengthening the Reporting of Observational Studies in Epidemiology.

**Table S2. Operational definitions and interpretive scope.**

| **Construct** | **Final operational definition** |
| --- | --- |
| Population universe | Adults aged 18–69 years in 37 included national or territorial STEPS settings with harmonizable sex, valid measured height/weight, positive Step 2 weight, complete PSU/stratum, and no known pregnancy. |
| Metabolic disease | Observed hypertension or diabetes; selected because these conditions were consistently harmonizable across rounds. |
| Operationalized NIH threshold | BMI ≥40 kg/m² or BMI 35.0–39.9 kg/m² with observed metabolic disease. |
| Operationalized ASMBS/IFSO threshold | BMI ≥35 kg/m² or BMI 30.0–34.9 kg/m² with observed metabolic disease. |
| Common classifiable universe | For paired NIH/ASMBS/IFSO comparison and component decomposition, records classifiable under ASMBS/IFSO. Adults with BMI 30.0–34.9 kg/m² and missing metabolic-disease information are excluded from the paired universe because they are ASMBS/IFSO-indeterminate. |
| NIH threshold stratum | Adults meeting the operationalized NIH threshold within the common ASMBS/IFSO-classifiable universe; identical to the NIH row in revised Table 2. |
| ASMBS/IFSO indeterminate | BMI 30.0–34.9 kg/m² with insufficient information to determine metabolic disease. Reported separately and not stacked as threshold attainment. |
| Primary projection | 2030 demographic stability: observed sex-by-age cell prevalence held constant and combined with WPP 2024 denominators. |
| High/exploratory projection | 2030 demographic plus epidemiological trend; interpreted conditionally and not as a forecast with complete uncertainty. |
| Clinical interpretation | Population-threshold attainment indicates a population for further assessment; it does not establish individual eligibility, candidacy, indication, contraindication status, preference, or utilization. |

Note. Abbreviations: ASMBS, American Society for Metabolic and Bariatric Surgery; BMI, body mass index; IFSO, International Federation for the Surgery of Obesity and Metabolic Disorders; NIH, National Institutes of Health; PSU, primary sampling unit; STEPS, STEPwise Approach to Noncommunicable Disease Risk Factor Surveillance; WPP, World Population Prospects.

**Table S3. Candidate survey inventory, inclusion decisions, and analytic roles.**

| **Setting** | **Year** | **WHO region** | **Primary round** | **Included** | **Analytic role** | **Exclusion or inclusion rationale** |
| --- | --- | --- | --- | --- | --- | --- |
| Afghanistan | 2018 | Eastern Mediterranean | TRUE | TRUE | Primary analysis | Passed all inclusion gates |
| Algeria | 2016 | Africa | TRUE | TRUE | Primary analysis | Passed all inclusion gates |
| Armenia | 2016 | Europe | TRUE | TRUE | Primary analysis | Passed all inclusion gates |
| Aruba | 2023 | Americas | TRUE | TRUE | Primary analysis | Passed all inclusion gates |
| Azerbaijan | 2017 | Europe | TRUE | TRUE | Primary analysis | Passed all inclusion gates |
| Bahamas | 2019 | Americas | TRUE | TRUE | Primary analysis | Passed all inclusion gates |
| Belarus | 2016 | Europe | TRUE | TRUE | Primary analysis | Passed all inclusion gates |
| Benin | 2015 | Africa | TRUE | TRUE | Primary analysis | Passed all inclusion gates |
| Bolivia (Plurinational State of) | 2019 | Americas | TRUE | TRUE | Primary analysis | Passed all inclusion gates |
| Brunei Darussalam | 2015 | Western Pacific | TRUE | TRUE | Primary analysis | Passed all inclusion gates |
| Burkina Faso | 2021 | Africa | TRUE | TRUE | Primary analysis | Passed all inclusion gates |
| Cabo Verde | 2020 | Africa | TRUE | TRUE | Primary analysis | Passed all inclusion gates |
| Central African Republic | 2017 | Africa | TRUE | FALSE | Excluded | Observed and documented survey age coverage is 25-64 years |
| Ecuador | 2018 | Americas | TRUE | TRUE | Primary analysis | Passed all inclusion gates |
| Ethiopia | 2015 | Africa | TRUE | TRUE | Primary analysis | Passed all inclusion gates |
| Georgia | 2016 | Europe | TRUE | TRUE | Primary analysis | Passed all inclusion gates |
| Guyana | 2016 | Americas | TRUE | TRUE | Primary analysis | Passed all inclusion gates |
| Iraq | 2015 | Eastern Mediterranean | TRUE | TRUE | Primary analysis | Passed all inclusion gates |
| Jordan | 2019 | Eastern Mediterranean | TRUE | TRUE | Primary analysis | Passed all inclusion gates |
| Kiribati | 2015 | Western Pacific | TRUE | TRUE | Primary analysis | Passed all inclusion gates |
| Lebanon - Lebanese population | 2017 | Eastern Mediterranean | TRUE | FALSE | Excluded | Survey population is not one-to-one with the total resident population in WPP |
| Lebanon - Syrian refugee population | 2017 | Eastern Mediterranean | TRUE | FALSE | Excluded | Refugee subpopulation cannot use the total Lebanon WPP denominator |
| Liberia | 2022 | Africa | TRUE | TRUE | Primary analysis | Passed all inclusion gates |
| Malawi | 2017 | Africa | TRUE | TRUE | Primary analysis | Passed all inclusion gates |
| Marshall Islands | 2017 | Western Pacific | TRUE | FALSE | Excluded | No verified Step 2 weight PSU or stratum in microdata |
| Micronesia (Federated States of) - Chuuk | 2016 | Western Pacific | TRUE | FALSE | Excluded | Subnational Chuuk survey cannot use the national WPP denominator |
| Mongolia | 2019 | Western Pacific | TRUE | TRUE | Primary analysis | Passed all inclusion gates |
| Morocco | 2017 | Eastern Mediterranean | TRUE | TRUE | Primary analysis | Passed all inclusion gates |
| Nauru | 2015 | Western Pacific | TRUE | FALSE | Excluded | Minimum classifiable age-sex cell n was 23 (<25) |
| Palau | 2016 | Western Pacific | TRUE | FALSE | Excluded | No verified Step 2 weight PSU or stratum in microdata |
| Republic of Moldova | 2021 | Europe | TRUE | TRUE | Primary analysis | Passed all inclusion gates |
| Saint Lucia | 2019 | Americas | TRUE | TRUE | Primary analysis | Passed all inclusion gates |
| Sao Tome and Principe | 2019 | Africa | TRUE | TRUE | Primary analysis | Passed all inclusion gates |
| Solomon Islands | 2015 | Western Pacific | TRUE | TRUE | Primary analysis | Passed all inclusion gates |
| Sudan | 2016 | Eastern Mediterranean | TRUE | TRUE | Primary analysis | Passed all inclusion gates |
| Tajikistan | 2016 | Europe | TRUE | TRUE | Primary analysis | Passed all inclusion gates |
| Tonga | 2017 | Western Pacific | TRUE | FALSE | Excluded | Comparable diabetes diagnosis and oral medication variables are structurally absent |
| Trinidad and Tobago | 2024 | Americas | TRUE | TRUE | Primary analysis | Passed all inclusion gates |
| Turkmenistan | 2018 | Europe | TRUE | TRUE | Primary analysis | Passed all inclusion gates |
| Tuvalu | 2015 | Western Pacific | TRUE | TRUE | Primary analysis | Passed all inclusion gates |
| Ukraine | 2019 | Europe | TRUE | TRUE | Primary analysis | Passed all inclusion gates |
| Uzbekistan | 2019 | Europe | TRUE | TRUE | Primary analysis | Passed all inclusion gates |
| Viet Nam | 2015 | Western Pacific | FALSE | FALSE | Temporal validation | Earlier repeat survey retained only for temporal validation |
| Viet Nam | 2021 | Western Pacific | TRUE | TRUE | Primary analysis | Passed all inclusion gates |
| Wallis and Futuna Islands | 2019 | Western Pacific | TRUE | TRUE | Primary analysis | Passed all inclusion gates |
| Zambia | 2017 | Africa | TRUE | TRUE | Primary analysis | Passed all inclusion gates |

Note. Forty-six candidate setting-year files were audited; 37 settings entered the primary cohort. WHO groupings are descriptive and do not imply regional representativeness. Detailed source metadata and restricted-data hashes are contained in the reproducibility package.

**Table S4. Reconstructed cohort flow and setting-level eligibility gates.**

| **Stage type** | **Stage** | **N retained** | **N excluded at stage** | **Reduction from prior retained N** | **Setting scope** |
| --- | --- | --- | --- | --- | --- |
| Retained | Candidate survey records | 174,542 | — | — | 46 candidate settings |
| Retained | Age 18-69 years | 172,836 | — | 1,706 | 46 candidate settings |
| Retained | Known binary sex | 172,836 | — | 0 | 46 candidate settings |
| Retained | Valid measured anthropometry | 158,811 | — | 14,025 | 46 candidate settings |
| Retained | Positive Step 2 survey weight | 154,695 | — | 4,116 | 46 candidate settings |
| Retained | Complete survey-design identifiers | 154,265 | — | 430 | 46 candidate settings |
| Excluded | Excluded owing to known pregnancy | — | 57 | — | 46 candidate settings |
| Retained | Individual analytic records after known-pregnancy exclusion (all candidate settings) | 154,208 | — | 57 | 46 candidate settings |
| Retained | Primary analytic cohort after setting-level eligibility gates | 138,695 | — | 15,513 | 37 included settings |

Note. The exact individual-level identity is 154,265 − 57 = 154,208. The subsequent reduction to 138,695 reflects setting-level gates and must not be represented as a single individual exclusion count.

**Table S5. Revised paired NIH and ASMBS/IFSO population-threshold estimates in the common classifiable universe.**

| **Scenario** | **Threshold or contrast** | **Adults** | **95% limits, adults** | **Prevalence or difference, %** | **95% limits, %** | **Interval classification** |
| --- | --- | --- | --- | --- | --- | --- |
| Baseline (survey-year population) | NIH 1991 threshold stratum | 13,358,000 | 12,778,000 to 14,022,000 | 3.63 | 3.48 to 3.82 | 95% design-based bootstrap percentile CI (stratified PSU resampling; conditional on fixed WPP denominators) |
| Baseline (survey-year population) | ASMBS/IFSO 2022 threshold | 38,127,000 | 37,058,000 to 39,240,000 | 10.37 | 10.08 to 10.68 | 95% design-based bootstrap percentile CI (stratified PSU resampling; conditional on fixed WPP denominators) |
| Baseline (survey-year population) | Increment beyond NIH threshold stratum | 24,770,000 | 23,914,000 to 25,671,000 | 6.74 | 6.51 to 6.98 | 95% design-based bootstrap percentile CI (stratified PSU resampling; conditional on fixed WPP denominators) |
| 2030 demographic stability (primary) | NIH 1991 threshold stratum | 17,070,000 | 16,358,000 to 17,892,000 | 3.65 | 3.49 to 3.82 | 95% design-based bootstrap percentile CI (stratified PSU resampling; conditional on fixed WPP denominators) |
| 2030 demographic stability (primary) | ASMBS/IFSO 2022 threshold | 48,549,000 | 47,275,000 to 49,849,000 | 10.37 | 10.10 to 10.65 | 95% design-based bootstrap percentile CI (stratified PSU resampling; conditional on fixed WPP denominators) |
| 2030 demographic stability (primary) | Increment beyond NIH threshold stratum | 31,478,000 | 30,521,000 to 32,518,000 | 6.72 | 6.52 to 6.95 | 95% design-based bootstrap percentile CI (stratified PSU resampling; conditional on fixed WPP denominators) |
| 2030 epidemiological trend (high/exploratory) | NIH 1991 threshold stratum | 23,415,000 | 22,285,000 to 24,599,000 | 5.00 | 4.76 to 5.25 | 95% conditional bootstrap percentile UI (stratified PSU resampling; conditional on fixed WPP/GHO projection inputs; not a full coverage CI) |
| 2030 epidemiological trend (high/exploratory) | ASMBS/IFSO 2022 threshold | 64,153,000 | 62,448,000 to 65,944,000 | 13.70 | 13.34 to 14.09 | 95% conditional bootstrap percentile UI (stratified PSU resampling; conditional on fixed WPP/GHO projection inputs; not a full coverage CI) |
| 2030 epidemiological trend (high/exploratory) | Increment beyond NIH threshold stratum | 40,738,000 | 39,537,000 to 42,004,000 | 8.70 | 8.45 to 8.97 | 95% conditional bootstrap percentile UI (stratified PSU resampling; conditional on fixed WPP/GHO projection inputs; not a full coverage CI) |

Note. Counts are displayed to the nearest 1,000; unrounded values are retained in the accompanying CSV. The paired ratio is 2.85 at survey-year baseline, 2.84 under 2030 demographic stability, and 2.74 in the high/exploratory scenario.

**Table S6. Mutually exclusive components of the operationalized ASMBS/IFSO population threshold.**

| **Scenario** | **Component** | **Adults** | **95% limits, adults** | **Interval classification** | **Role** |
| --- | --- | --- | --- | --- | --- |
| Baseline (survey-year population) | NIH 1991 threshold stratum | 13,358,000 | 12,778,000 to 14,022,000 | 95% design-based bootstrap percentile CI (stratified PSU resampling; conditional on fixed WPP denominators) | Mutually exclusive component of the ASMBS/IFSO threshold; not a clinical candidacy category |
| Baseline (survey-year population) | BMI 35.0-39.9, not in NIH threshold stratum | 3,879,000 | 3,607,000 to 4,173,000 | 95% design-based bootstrap percentile CI (stratified PSU resampling; conditional on fixed WPP denominators) | Mutually exclusive component of the ASMBS/IFSO threshold; not a clinical candidacy category |
| Baseline (survey-year population) | BMI 30.0-34.9 with observed metabolic disease | 20,891,000 | 20,055,000 to 21,722,000 | 95% design-based bootstrap percentile CI (stratified PSU resampling; conditional on fixed WPP denominators) | Mutually exclusive component of the ASMBS/IFSO threshold; not a clinical candidacy category |
| Baseline (survey-year population) | ASMBS/IFSO total (closure check) | 38,127,000 | 37,058,000 to 39,240,000 | 95% design-based bootstrap percentile CI (stratified PSU resampling; conditional on fixed WPP denominators) | Sum of the three mutually exclusive operationalized threshold components |
| 2030 demographic stability (primary) | NIH 1991 threshold stratum | 17,070,000 | 16,358,000 to 17,892,000 | 95% design-based bootstrap percentile CI (stratified PSU resampling; conditional on fixed WPP denominators) | Mutually exclusive component of the ASMBS/IFSO threshold; not a clinical candidacy category |
| 2030 demographic stability (primary) | BMI 35.0-39.9, not in NIH threshold stratum | 4,944,000 | 4,556,000 to 5,342,000 | 95% design-based bootstrap percentile CI (stratified PSU resampling; conditional on fixed WPP denominators) | Mutually exclusive component of the ASMBS/IFSO threshold; not a clinical candidacy category |
| 2030 demographic stability (primary) | BMI 30.0-34.9 with observed metabolic disease | 26,535,000 | 25,602,000 to 27,482,000 | 95% design-based bootstrap percentile CI (stratified PSU resampling; conditional on fixed WPP denominators) | Mutually exclusive component of the ASMBS/IFSO threshold; not a clinical candidacy category |
| 2030 demographic stability (primary) | ASMBS/IFSO total (closure check) | 48,549,000 | 47,275,000 to 49,849,000 | 95% design-based bootstrap percentile CI (stratified PSU resampling; conditional on fixed WPP denominators) | Sum of the three mutually exclusive operationalized threshold components |
| 2030 epidemiological trend (high/exploratory) | NIH 1991 threshold stratum | 23,415,000 | 22,285,000 to 24,599,000 | 95% conditional bootstrap percentile UI (stratified PSU resampling; conditional on fixed WPP/GHO projection inputs; not a full coverage CI) | Mutually exclusive component of the ASMBS/IFSO threshold; not a clinical candidacy category |
| 2030 epidemiological trend (high/exploratory) | BMI 35.0-39.9, not in NIH threshold stratum | 7,418,000 | 6,985,000 to 7,985,000 | 95% conditional bootstrap percentile UI (stratified PSU resampling; conditional on fixed WPP/GHO projection inputs; not a full coverage CI) | Mutually exclusive component of the ASMBS/IFSO threshold; not a clinical candidacy category |
| 2030 epidemiological trend (high/exploratory) | BMI 30.0-34.9 with observed metabolic disease | 33,320,000 | 32,143,000 to 34,378,000 | 95% conditional bootstrap percentile UI (stratified PSU resampling; conditional on fixed WPP/GHO projection inputs; not a full coverage CI) | Mutually exclusive component of the ASMBS/IFSO threshold; not a clinical candidacy category |
| 2030 epidemiological trend (high/exploratory) | ASMBS/IFSO total (closure check) | 64,153,000 | 62,448,000 to 65,944,000 | 95% conditional bootstrap percentile UI (stratified PSU resampling; conditional on fixed WPP/GHO projection inputs; not a full coverage CI) | Sum of the three mutually exclusive operationalized threshold components |

Note. Within each scenario, the three components sum exactly to the ASMBS/IFSO total in source values. The NIH threshold stratum is identical to the NIH total in Table S5 because both use the common classifiable universe.

**Table S7. NIH component reconciliation and denominator diagnosis.**

| **Scenario or diagnostic** | **Original NIH support / records** | **Common-universe NIH** | **Revised Table 2 NIH** | **Difference from component** | **Resolution or interpretation** |
| --- | --- | --- | --- | --- | --- |
| Baseline (survey-year population) | 13,291,572 | 13,357,666 | 13,357,666 | 0 | Table 2 revision uses the common-universe NIH component; it closes exactly with the component stack. The original full-support NIH value is retained for audit only. |
| 2030 demographic stability (primary) | 16,968,912 | 17,070,353 | 17,070,353 | 0 | Table 2 revision uses the common-universe NIH component; it closes exactly with the component stack. The original full-support NIH value is retained for audit only. |
| 2030 epidemiological trend (high/exploratory) | 23,280,086 | 23,414,746 | 23,414,746 | 0 | Table 2 revision uses the common-universe NIH component; it closes exactly with the component stack. The original full-support NIH value is retained for audit only. |
| Primary analytic records | 138,695 | — | — | — | Restricted records only; microdata are not exported. |
| NIH=0 but component_nih is missing | 222 | — | — | — | These records are determinately negative for NIH but not classifiable for ASMBS/IFSO. |
| BMI 30.0-34.9 with metabolic disease missing | 222 | — | — | — | This is the structural source of the differing calculation denominators. |
| ASMBS/IFSO indeterminate | 222 | — | — | — | Reported separately; never inserted into the eligible component stack. |

Note. The previous discrepancy was caused by different classification support, not arithmetic error. Adults with BMI 30.0–34.9 kg/m² and missing metabolic-disease information are determinate negatives for NIH but indeterminate for ASMBS/IFSO. Revised paired tables use the common classifiable universe and therefore reconcile exactly.

**Table S8. Coverage of the six World Health Organization regions by included settings.**

| **WHO region** | **Included settings** | **Included population, ages 18–69** | **WHO-region population, ages 18–69** | **Coverage, %** | **Interpretive restriction** |
| --- | --- | --- | --- | --- | --- |
| Africa | 9 | 167,932,742 | 773,183,902 | 21.7 | Partial, non-probability setting coverage: describe included settings only; do not infer to the WHO region or globally. |
| Americas | 7 | 23,308,875 | 722,668,650 | 3.2 | Partial, non-probability setting coverage: describe included settings only; do not infer to the WHO region or globally. |
| Eastern Mediterranean | 5 | 120,690,804 | 546,895,806 | 22.1 | Partial, non-probability setting coverage: describe included settings only; do not infer to the WHO region or globally. |
| Europe | 9 | 82,379,160 | 620,908,967 | 13.3 | Partial, non-probability setting coverage: describe included settings only; do not infer to the WHO region or globally. |
| South-East Asia | 0 | 0 | 1,292,058,658 | 0.0 | No included setting: no WHO-region estimate may be made. |
| Western Pacific | 7 | 73,864,168 | 1,549,511,574 | 4.8 | Partial, non-probability setting coverage: describe included settings only; do not infer to the WHO region or globally. |

Note. Coverage quantifies external validity only. Included settings were not selected probabilistically from WHO regions; grouped estimates must not be interpreted as complete regional or global burden.

**Table S9. Asian-threshold sensitivity using prespecified M49 geographic proxies.**

| **Definition** | **Settings affected** | **Sensitivity adults** | **Difference, adults** | **Sensitivity prevalence, %** | **Difference, percentage points** | **Interval classification** | **Scope limitation** |
| --- | --- | --- | --- | --- | --- | --- | --- |
| Narrow prespecified M49 subregional proxy | 4 | 56,952,000 | 8,403,000 | 12.16 | 1.79 | Point sensitivity only; no separate interval is estimated. | Geographic M49 sensitivity limited to Eastern, South-eastern and Southern Asia. It does not identify ethnicity and does not imply clinical eligibility/candidacy. |
| Broad all-M49 Asia boundary analysis (exploratory) | 12 | 76,456,000 | 27,908,000 | 16.33 | 5.96 | Point sensitivity only; no separate interval is estimated. | Broad geographic M49 sensitivity across settings located in Asia. It is deliberately exploratory, does not identify ethnicity, and must not be interpreted as a clinical threshold recommendation. |

Note. The narrower proxy includes M49 Eastern, South-eastern, or Southern Asia. The broad boundary analysis includes all settings geographically classified in Asia by M49, including Western and Central Asia. M49 geography is not an ethnic classification, and neither analysis determines clinical eligibility or candidacy.

**Table S10. Interval terminology and propagated uncertainty sources.**

| **Estimand** | **Reported term** | **Basis** | **Prohibited claim** |
| --- | --- | --- | --- |
| Survey-year and 2030 demographic threshold estimates | 95% design-based bootstrap percentile CI | Stratified PSU bootstrap of survey estimates, conditional on fixed WPP denominators. | Not an interval for unobserved settings or any WHO region. |
| 2030 epidemiological-trend threshold estimates | 95% conditional bootstrap percentile UI | Bootstrap survey variation conditional on fixed WPP/GHO calibration and model choices. | Do not label this UI a complete CI for projected regional/global population burden. |
| Common-universe NIH/ASMBS paired difference and ratio | 95% design-based bootstrap percentile CI for baseline/demographic; conditional UI for high trend | Paired bootstrap preserves within-replicate covariance; the label follows the scenario type. | Do not generalize the paired comparison beyond the 37 included settings. |
| Shapley decomposition of 2030 ASMBS/IFSO change | 95% conditional bootstrap percentile UI | Bootstrap variation conditional on GHO/WPP inputs, model selection and included settings. | Do not call Shapley UI a causal CI or a WHO-region CI. |
| Asian M49 geographic sensitivity | No interval; point sensitivity | Changing M49 geographic scope is a deterministic scenario comparison; ethnic and clinical uncertainty are not represented. | Do not call this a CI or infer ethnicity or clinical candidacy. |

Note. Design-based confidence intervals and conditional uncertainty intervals are intentionally distinguished. Neither supports inference to unsampled WHO-region populations or to the world.

**Table S11. Reanalysis execution and acceptance controls.**

| **Control** | **Acceptance test** | **Observed result** | **Status** |
| --- | --- | --- | --- |
| QA-01 | Cohort flow retained counts are monotonic | Retained progression: 174,542 -> 172,836 -> 172,836 -> 158,811 -> 154,695 -> 154,265 -> 154,208 -> 138,695 | PASS |
| QA-02 | Known-pregnancy exclusion identity | 154265 retained before pregnancy exclusion - 57 excluded = 154208 retained afterwards. | PASS |
| QA-03 | Cohort flow has no negative removals | records_removed_from_prior is defined only for retained stages and is non-negative at every defined stage. | PASS |
| QA-04 | Pregnancy exclusion row is not a retained total | The exclusion event has records_excluded only; records_retained and records_removed_from_prior are blank. | PASS |
| QA-05 | Source and delivery directories separated | Source: G:/Mi unidad/Manuscritos/01_Articulos enviados a publicar/WHO_Bariatrica/ANALISIS_CIERRE_REAL_2026-07-24 | PASS |
| QA-06 | Corrected Table 2 NIH equals revised component NIH | Maximum absolute revised Table 2/component difference = 0 adults | PASS |
| QA-07 | Integer component stack closes to ASMBS/IFSO total | Maximum absolute integer closure error = 0 adults | PASS |
| QA-08 | Source Shapley decomposition closes | Maximum absolute source Shapley error = 0; declared tolerance <1e-8 | PASS |
| QA-09 | Representativeness table contains all six WHO regions | Regions: Africa; Americas; Eastern Mediterranean; Europe; South-East Asia; Western Pacific | PASS |
| QA-10 | No regional inference statement is emitted | All regions have partial or zero setting coverage. | PASS |
| QA-11 | M49 sensitivity explicitly separated from ethnicity/clinical inference | Tables and Figure 3 identify M49 geography only and state the interpretive prohibition. | PASS |
| QA-12 | GPU availability documented; no unvalidated GPU acceleration claimed | GPU probe: NVIDIA GeForce RTX 5060, 610.88, 8151 MiB ; cupy/cudf/torch unavailable in the validated Python environment; survey bootstrapping remains CPU. | PASS |

Note. All 12 analytic controls passed. Five additional Word-level controls for exact flow identity, explicit exclusion count, terminology, encoding, and document integrity also passed in the validated traceability report.

**Table S12. Summary of additional robustness and validation analyses.**

| **Analysis** | **Result** | **Interpretive restriction** |
| --- | --- | --- |
| Age 20–69 years | Prevalence +0.54 percentage points | Different population universe; counts not compared. |
| Jeffreys smoothing | Prevalence +0.15 percentage points; adults +699,679 | Same universe; small-cell sensitivity. |
| Viet Nam 2015 instead of 2021 | Prevalence +0.02 percentage points; adults +98,584 | Not interpreted as a causal pandemic effect. |
| Regional trend backup | Adults −3,000 | Negligible aggregate change. |
| Step 3 biomarkers | Prevalence +0.32 percentage points; adults +838,449 | Paired subset with verified biomarker weights and denominators. |
| Leave-one-setting-out | Difference ranged from −1.15 to +2.03 percentage points | Descriptive influence analysis. |
| All indeterminate as not meeting threshold | Prevalence −0.08 percentage points; adults −356,888 | Extreme lower bound. |
| All indeterminate as meeting threshold | Prevalence +0.24 percentage points; adults +1,131,298 | Extreme upper bound. |
| Inverse-probability weighting for classifiability | No change in primary estimate | Classification-weighting sensitivity. |

Note. Detailed machine-readable outputs and executable scripts are included in the reproducibility package.

**Table S13. Setting-level point estimates in the 2030 demographic-stability scenario, common classifiable universe.**

| **Setting** | **WHO region** | **NIH threshold, %** | **NIH adults** | **ASMBS/IFSO threshold, %** | **ASMBS/IFSO adults** | **ASMBS/IFSO indeterminate, %** |
| --- | --- | --- | --- | --- | --- | --- |
| Algeria | Africa | 4.8 | 1,505,188 | 15.2 | 4,735,989 | 0.1 |
| Benin | Africa | 1.6 | 141,014 | 5.0 | 440,441 | 0.0 |
| Burkina Faso | Africa | 1.4 | 205,341 | 4.5 | 650,121 | 0.0 |
| Cabo Verde | Africa | 3.4 | 12,530 | 10.4 | 38,395 | 0.1 |
| Ethiopia | Africa | 0.3 | 232,793 | 1.0 | 753,025 | 0.0 |
| Liberia | Africa | 7.1 | 244,618 | 15.3 | 527,423 | 0.1 |
| Malawi | Africa | 0.9 | 123,402 | 2.9 | 396,802 | 0.0 |
| Sao Tome and Principe | Africa | 3.2 | 4,640 | 9.1 | 13,179 | 0.0 |
| Zambia | Africa | 1.9 | 247,752 | 5.6 | 740,270 | 0.0 |
| Aruba | Americas | 13.8 | 10,040 | 30.9 | 22,503 | 0.0 |
| Bahamas | Americas | 20.4 | 59,194 | 35.2 | 102,050 | 0.0 |
| Bolivia (Plurinational State of) | Americas | 5.3 | 445,896 | 14.6 | 1,225,089 | 0.0 |
| Ecuador | Americas | 4.5 | 570,149 | 15.7 | 1,994,837 | 0.0 |
| Guyana | Americas | 8.0 | 42,562 | 18.3 | 97,535 | 0.0 |
| Saint Lucia | Americas | 12.0 | 15,874 | 25.4 | 33,630 | 0.0 |
| Trinidad and Tobago | Americas | 10.9 | 115,800 | 24.8 | 262,988 | 0.0 |
| Afghanistan | Eastern Mediterranean | 4.6 | 1,186,078 | 13.3 | 3,404,050 | 0.0 |
| Iraq | Eastern Mediterranean | 11.3 | 3,407,314 | 27.0 | 8,161,824 | 4.5 |
| Jordan | Eastern Mediterranean | 9.3 | 732,702 | 23.0 | 1,810,438 | 0.0 |
| Morocco | Eastern Mediterranean | 3.7 | 978,286 | 13.2 | 3,465,813 | 0.0 |
| Sudan | Eastern Mediterranean | 2.8 | 877,905 | 8.1 | 2,516,893 | 0.0 |
| Armenia | Europe | 7.3 | 139,720 | 18.9 | 361,812 | 0.0 |
| Azerbaijan | Europe | 5.8 | 432,551 | 16.5 | 1,236,343 | 0.3 |
| Belarus | Europe | 7.4 | 440,667 | 22.4 | 1,332,527 | 0.0 |
| Georgia | Europe | 11.6 | 286,393 | 26.8 | 660,182 | 0.1 |
| Republic of Moldova | Europe | 4.4 | 81,016 | 17.4 | 322,858 | 0.0 |
| Tajikistan | Europe | 4.6 | 310,619 | 14.7 | 993,717 | 0.0 |
| Turkmenistan | Europe | 3.8 | 195,420 | 11.9 | 609,264 | 0.0 |
| Ukraine | Europe | 7.7 | 2,085,197 | 20.9 | 5,635,000 | 0.2 |
| Uzbekistan | Europe | 6.5 | 1,555,907 | 19.7 | 4,720,259 | 0.1 |
| Brunei Darussalam | Western Pacific | 8.6 | 29,562 | 22.4 | 77,109 | 0.0 |
| Kiribati | Western Pacific | 14.4 | 12,348 | 32.0 | 27,380 | 0.0 |
| Mongolia | Western Pacific | 4.2 | 96,508 | 15.5 | 357,108 | 0.0 |
| Solomon Islands | Western Pacific | 4.5 | 23,953 | 11.8 | 63,232 | 0.1 |
| Tuvalu | Western Pacific | 25.4 | 1,320 | 47.7 | 2,476 | 0.2 |
| Viet Nam | Western Pacific | 0.3 | 217,903 | 1.1 | 752,471 | 0.0 |
| Wallis and Futuna Islands | Western Pacific | 29.6 | 2,190 | 50.6 | 3,740 | 0.0 |

Note. ASMBS/IFSO point prevalence and adults are exact sums of the three mutually exclusive component point estimates. Joint setting-level intervals are not reconstructed by summing component limits and are therefore not reported in this compact table. These settings do not represent complete WHO regions.

**Table S14. Age- and sex-specific 2030 demographic-stability estimates.**

| **Sex and age group** | **Projected prevalence, % (95% design-based CI)** | **Projected adults (95% design-based CI)** |
| --- | --- | --- |
| Men, 18-29 years | 2.8 (2.3 to 3.3) | 2,185,574 (1,850,035 to 2,575,576) |
| Men, 30-44 years | 7.1 (6.5 to 7.7) | 5,594,755 (5,097,496 to 6,080,316) |
| Men, 45-59 years | 12.1 (11.1 to 13.1) | 6,415,388 (5,889,458 to 6,932,651) |
| Men, 60-69 years | 13.7 (12.7 to 14.7) | 2,970,505 (2,755,867 to 3,187,429) |
| Women, 18-29 years | 4.3 (3.8 to 4.9) | 3,329,536 (2,937,273 to 3,757,671) |
| Women, 30-44 years | 12.4 (11.8 to 13.0) | 9,664,808 (9,208,680 to 10,130,107) |
| Women, 45-59 years | 21.6 (20.9 to 22.5) | 11,988,940 (11,577,246 to 12,469,085) |
| Women, 60-69 years | 25.2 (24.0 to 26.5) | 6,399,270 (6,089,725 to 6,722,533) |

Note. These strata are descriptive. Formal interaction terms were not estimated. Abbreviation: CI, confidence interval.

**Supplementary figures**

**Figure S1. Reconstructed flow from candidate records to the final primary cohort.**

Note. The diagram separates retained counts from exclusions and preserves the exact identity 154,265 − 57 = 154,208. STEPS, STEPwise Approach to Noncommunicable Disease Risk Factor Surveillance.

**Figure S2. Population coverage of the six World Health Organization regions by included settings.**

Note. Coverage describes the represented proportion of each World Health Organization (WHO) region population aged 18–69 years in 2030. The included settings were not sampled to support regional or global inference.

**Figure S3. Setting-level prevalence of meeting the operationalized NIH 1991 and ASMBS/IFSO 2022 thresholds in the 2030 demographic-stability scenario.**

Note. Estimates use the common classifiable universe and are descriptive for the 37 included settings. ASMBS, American Society for Metabolic and Bariatric Surgery; IFSO, International Federation for the Surgery of Obesity and Metabolic Disorders; NIH, National Institutes of Health.

**Figure S4. Internally selected 2015 Global Health Observatory obesity-trend fits and extrapolation by setting.**

**Panel A.**

**Figure S4 (continued). Panel B.**

**Figure S4 (continued). Panel C.**

**Figure S4 (continued). Panel D.**

Note. The 2015 window was selected by internal rolling-origin performance but could not be directly validated against the repeated STEPS survey. Curves are extrapolations under the high/exploratory scenario, not forecasts with complete uncertainty. GHO, Global Health Observatory; STEPS, STEPwise Approach to Noncommunicable Disease Risk Factor Surveillance; WHO, World Health Organization.

**Figure S5. Model-governance hierarchy and direct-validation error.**

Note. The demographic benchmark had 8.0% direct error; the best directly testable epidemiological specification had 37.4%. The full high path and internally selected 2015 window were not directly testable in the repeated-round comparison. BMI, body mass index; GHO, Global Health Observatory; STEPS, STEPwise Approach to Noncommunicable Disease Risk Factor Surveillance; WHO, World Health Organization.

**Figure S6. Differences from the primary demographic-stability estimate across paired and alternative sensitivity analyses.**

Note. Bars are point differences in prevalence relative to the primary operationalized ASMBS/IFSO estimate; analyses that change the population universe are interpreted separately. The paired Step 3 biomarker sensitivity is included among the displayed analyses. ASMBS, American Society for Metabolic and Bariatric Surgery; BMI, body mass index; GHO, Global Health Observatory; IFSO, International Federation for the Surgery of Obesity and Metabolic Disorders; M49, United Nations Standard Country or Area Codes for Statistical Use; WHO, World Health Organization; WPP, World Population Prospects.

**Figure S7. Asian-threshold sensitivity under narrow and broad M49 geographic definitions.**

Note. M49 is a statistical geographic classification. The broad analysis is exploratory and does not imply ethnicity, setting-wide clinical applicability, eligibility, or individual candidacy. ASMBS, American Society for Metabolic and Bariatric Surgery; BMI, body mass index; IFSO, International Federation for the Surgery of Obesity and Metabolic Disorders; M49, United Nations Standard Country or Area Codes for Statistical Use.

**Figure S8. Leave-one-setting-out influence on the aggregate 2030 demographic-stability prevalence.**

Note. Values show the change in aggregate prevalence after omitting one setting. The analysis is descriptive and does not quantify a causal effect or regional representativeness. ASMBS, American Society for Metabolic and Bariatric Surgery; IFSO, International Federation for the Surgery of Obesity and Metabolic Disorders.

**Figure S9. Missing-classification and minimum-cell diagnostics by included setting.**

Note. The diagnostic displays NIH-indeterminate status, ASMBS/IFSO-indeterminate status, and the minimum classifiable sex-by-age cell size for each included setting. ASMBS, American Society for Metabolic and Bariatric Surgery; IFSO, International Federation for the Surgery of Obesity and Metabolic Disorders; NIH, National Institutes of Health.
